# Evaluation of a Commercial Pregnancy Test Using Blood or Plasma Samples in High-Producing Dairy Cows

**DOI:** 10.3390/ani14111656

**Published:** 2024-05-31

**Authors:** Fernando López-Gatius, Sergi Ganau, Irina Garcia-Ispierto

**Affiliations:** 1 Agrotecnio Centre, 25198 Lleida, Spain; irina.garcia@udl.cat; 2 Transfer in Bovine Reproduction SLu, 22300 Barbastro, Spain; 3 Granja Sant Josep, La Melusa, Tamarite, 22549 Huesca, Spain; 4 Department of Animal Science, University of Lleida, 25198 Lleida, Spain

**Keywords:** early nonpregnancy diagnosis, embryonic loss, milk yield, on-farm pregnancy tests, pedometers, postpartum PAGs, rapid pregnancy tests

## Abstract

**Simple Summary:**

Early pregnancy diagnosis is of major importance to the economics of dairy herds. The detection of placental proteins, termed pregnancy-associated glycoproteins (PAGs), represents a diagnostic tool to identify both pregnant and non-pregnant cows. However, as milk production correlates negatively with plasma levels of PAGs during early pregnancy, detection of PAGs may be hindered by high milk production. This study evaluates a newly developed commercial PAGs-based pregnancy test using whole blood or plasma samples during early pregnancy (28–55 days of gestation) in high-producing dairy cows.

**Abstract:**

This study evaluated a commercial pregnancy-associated glycoproteins (PAGs)-based pregnancy test using whole blood or plasma samples during early pregnancy (28–55 days of gestation) in high-producing dairy cows. Transrectal ultrasonography was used as the gold standard method. The study population constituted of 284 cows. False positive diagnoses were recorded from Day 60 to 89 and from Day 60 to 99 postpartum in blood and plasma samples, respectively. In early pregnancy screening, correct positive diagnoses were recorded in 75% and 100% of blood and plasma samples, respectively. High milk production was associated with negative results in blood samples and with the lowest test line intensity in plasma samples. False positive or negative diagnoses were recorded in 0% of both types of samples in cows previously diagnosed as pregnant and showing signs of estrus. In conclusion, the use of plasma was more effective than the use of blood in early pregnancy diagnosis. In cows previously diagnosed as pregnant and showing signs of estrus, both types of samples showed the same results. Because of large individual variations, normal single pregnancies could not be differentiated from twin pregnancies, from pregnancies with a recently dead conceptus, or from pregnancies that experienced subsequent pregnancy loss.

## 1. Introduction

As early as 1924, high milk production has been positively correlated with improved fertility in dairy cattle [1]. More recently, the combination of high fertility and high production has been linked to cow health as a consequence of good feeding, housing, and management practices on the herd [2,3,4,5]. In effect, high-producing herds show better fertility than low-producing herds [6,7,8,9,10]. Even at the present time and after decades of fertility decline, recent changes in genetic selection for fertility and improvements in healthcare and cow comfort have led to the ongoing increase of reproductive efficiency [11,12]. In addition, advancements in cow welfare at both individual and herd levels have been notable in the past two decades, potentially alleviating societal concerns regarding dairy production [12,13]. The general public is also calling for reductions in the environmental footprint. Adequate herd-level reproductive programs not only bring increased milk output on the herds but also make the use of land resources more efficient, thus reducing greenhouse gas emissions [14,15,16]. In fact, good reproductive performance reduces the generation interval and, thus, hastens genetic gain for efficient feed use, fertility, and low CO2-equivalent emissions [16]. In this context, intensive reproductive control meets the needs of today’s management practices in dairy herds. A technique to be refined is the early nonpregnancy diagnosis of inseminated cows in order to allow prompt re-insemination of the cow where necessary.

Pregnancy is a crucial event in the life of the dairy cow, encompassing the embryonic period from conception to around 45 days of gestation and continuing through the fetal period until parturition [17]. Following a positive pregnancy diagnosis during the late embryonic period, there is up to 20% pregnancy loss, which is drastically reduced after Day 60 [18,19,20], when gestation is firmly established [21,22]. This is the reason why most clinical efforts to diagnose pregnancy and pregnancy viability are made during the late embryonic/early fetal period, between days 22 and 60 of gestation [18,19,20]. During this period, ultrasound is the gold standard method for diagnosing pregnancy and for predicting pregnancy viability or failure [18,20,23]. However, detection of pregnancy-associated glycoproteins (PAGs) of trophoblastic origin is emerging as an alternative or a support to ultrasound pregnancy controls in cattle [18,19,24].

The presence of PAGs in maternal blood was first described in 1982 [25]. Placental PAGs were purified [26] and proposed as markers for pregnancy diagnosis ten years later [27]. In pregnant cows, PAGs can be detected from 15 to 22 days post-AI [27,28,29] and progressively increase their values from Day 60 of gestation up to 5–10 days before parturition [28,29,30]. After this time point, blood levels rapidly decrease up to parturition and during the postpartum period [29,30]. However, substantial concentrations of PAGs can be detected 80–100 days post-partum, such that this type of test should be performed from this date [29,31,32]. Due to the high individual variation in the presence of these proteins during the early gestation, tests for pregnancy diagnosis are furthermore recommended to be performed from Day 28 onwards [33,34]. In addition, results obtained during the late embryonic period can be masked under certain circumstances. Cows suffering subsequent pregnancy loss show significantly reduced PAGs values [35,36,37], whereas cows carrying twins have frequently higher circulating concentrations of PAGs [30,33,38,39]. Finally, milk production can also influence detection of PAGs. Milk production correlates negatively with plasma levels of PAGs during the early fetal period in high-producing dairy cows with live fetuses [40].

Pregnancy tests for the detection of PAGs have been assessed in the field using maternal plasma [41,42,43], serum [44,45], whole blood [44,46], or milk [41,42,44,45,46,47] samples. Although these tests may be useful in confirming pregnancy at the herd level, the timing of the test may influence the diagnosis of non-pregnancy [29,31,32]. The focus has been on the post-AI period of the test, without considering the possible presence of residual postpartum PAGs [41,42,43,44,45,46,47]. As a result, tests performed before Day 100 postpartum may give false positives [29,31,32]. Based on recent evidence of laboratory trials, and non-cross-reaction with other hormones, we hypothesized that the current test presents greater sensitivity compared with other tests. The current study was designed to evaluate a commercial PAGs-based pregnancy test, not previously assessed in field trials, using blood or plasma samples during the late embryonic period (28–34 days of gestation) or the early fetal period (49–55 days of gestation) in high-producing dairy cows. A second objective was to check the presence of PAGs in cows previously diagnosed as pregnant and showing estrous signs from Day 40 to 200 post-AI. Transrectal ultrasonography was performed for pregnancy diagnosis 2–24 h before the test as the gold standard. The sensitivity and specificity of the test was also analyzed.

## 2. Materials and Methods

### 2.1. Cows and Herd Management

This prospective observational cohort study was conducted in a Holstein high-producing dairy herd in north-eastern Spain (latitude 41.13 N, longitude −2.4 E). During the study period, from November 2023 to April 2024, the mean number of lactating cows in the herd was 4347, with a mean culling rate of 23.2% and daily milk production of 46.8 kg per milking cow. The cows were milked thrice daily and fed complete rations, which were the same from Day 30 post-partum until dry-off. Lactating cows were grouped according to number of pregnancies into primiparous, secundiparous, or cows in their third lactation or more. Dry cows were kept separately and were marked as the “parturition group” 7–25 days before the expected date of calving, depending on whether or not they were carrying twins. The cows were equipped with a radio-telemetric pedometer, such that walking activity values were recorded at the milking parlor at every milking, transferred to a desktop computer, and analyzed automatically using a herd management computer program (AfiFarm System; Afikim, Israel). Walking activity greater than 90% of the mean activity recorded in the previous ten days was taken as the lower limit for a cow to be considered in estrus. If estrus was suspected using the pedometer system, it was confirmed by rectal palpation of the cervix and vagina to obtain vaginal fluid and the animals were inseminated. The voluntary waiting period was 60 days for primiparous cows and 50 days for multiparous cows. All cows were artificially inseminated for the first time before 120 days in lactation. Eighty-two percent of all inseminations were performed at spontaneous estrus. The herd was maintained on a weekly reproductive health program. 

Only healthy cows were included in the study, as indicated by a body condition score of 2–3.5 on a scale of 1 to 5 [48], plus absence of clinical signs of disease at the time of the test. Exclusion criteria were the following pathologies: mastitis, lameness, digestive disorders, and pathological abnormalities of the reproductive tract detectable by ultrasonography. All animals were reared within the herd.

### 2.2. Pregnancy Diagnosis

All gynecological exams were performed by transrectal ultrasonography using a portable B-mode ultrasound scanner equipped with a 5–10 MHz transducer (E.I. Medical IBEX LITE; E.I. Medical Imaging, Loveland, CO, USA). Scanning was performed along the dorso/lateral surface of each uterine horn for pregnancy diagnosis on all animals. Pregnancy was diagnosed 28–34 days post-AI and assessed at 49–55 and 77–82 days post-AI. The presence of twins and a dead embryo or fetus were recorded. All gynecological exams and pregnancy diagnoses were performed by the same operator.

### 2.3. Testing Whole Blood and Plasma Samples for PAGs

All cows were sampled for whole blood and plasma. With regard to the ethical aspects, blood sampling was performed in accordance with good veterinary practices and approved by the Animal Ethics Committee of the University of Lleida, Faculty of Veterinary Medicine, Spain. Blood samples were withdrawn from the coccygeal artery or vein of all animals 2–24 h after the pregnancy diagnosis into two ethylenediamine tetra-acetic acid (EDTA)-coated vacuum tubes (Ring Biotechnology Co., Ltd., Beijing, China). Plasma was obtained from one of the tubes by centrifugation (2000× *g* for 10 min). Both whole blood and plasma were assayed on the farm within 30 min of collection. 

Prior to commercial distribution, each batch of kits is validated in the company’s laboratory (Ring Biotechnology Co., Ltd., Beijing, China) for possible interference with other blood substances and for the minimum detection level of PAGs. Bovine serum albumin, bovine immune-gamma globulin G (IgG), bovine casein, progesterone, estradiol, luteinizing hormone (LH), and follicle stimulating hormone (FSH) have no cross-reactions with the test kit, thus they will not interfere with the test result. The minimum detection level of this kit is set as 1.0 ng/mL PAGs, which is the cut-off value obtained based on former research, from 0.8 ng/mL to 1.5 ng/mL [49,50,51]. The mouse anti-PAG monoclonal antibody is coated, which was produced by purified PAG injected into balb/c mouse. In this test kit, two antibodies are used, one is for conjugate, the other is coated on the test line. The tests were performed according the manufacturer’s instructions by a veterinarian blinded to the gynecological status of the cows.

The blood test was performed by adding 40 μL of whole blood plus 60 μL of sample buffer to the well of a PAG antibody-coated lateral flow immunoassay plate (Ringbio Bovine Pregnancy Rapid Test Kit; Ring Biotechnology Co., Ltd., Beijing, China). For the plasma test, 120 μL of plasma was added to the test well. The plates with blood or plasma were incubated at room temperature (20–22 °C) for 15 min. At the end of the incubation period, the presence of two lines indicated pregnancy and the presence of a single line indicated non-pregnancy.

### 2.4. Experimental Design

The test was adapted to the routine practices and reproductive parameters of the herd.

#### 2.4.1. The Postpartum Period

To prevent the risk of false positives during the early postpartum period [29,31,32], a cut-off was determined for days in milk (DIM). Samples from ten non-inseminated cows were taken for each ten-day period from Day 60 to 119 postpartum (Table 1: *n* = 120 samples). The cut-off was taken to check when, in the cows’ DIM, all the samples were 100% negative.

#### 2.4.2. Pregnancy Diagnosis 

Within the weekly visit, primiparous, secundiparous, or cows in their third lactation or more diagnosed pregnant 28–34 days (*n* = 100 cows) or confirmed pregnant 49–55 days (*n* = 100 cows) post-AI were balanced to be included in the study. Samples were taken two hours after pregnancy diagnosis (primiparous and secundiparous cows) or 24 h later (cows in their third lactation or more) from the first diagnosed pregnancies the day of the visit. Pregnancies were classified as normal (*n* = 185 cows): carrying live singletons (*n* = 157) or twins (*n* = 28), or impending failure: cows carrying a recent dead singleton (*n* = 15). The absence of a heartbeat of the embryo or fetus within its amnion was considered a sign of recent death of the conceptus and recorded as a impending pregnancy loss. One pregnant cow bearing disorganized remnants of the conceptus 49 days post-AI was not included in the study. Two pregnant cows carrying triplets were also excluded from the study.

Data derived from the herd during the 12 months before the beginning of the study showed a 9.5% of pregnancy loss before Day 60 of gestation in pregnant cows with at least a live embryo at pregnancy diagnosis (Days 28–34 post-AI). Therefore, a cut-off of pregnancy loss before 60 days of pregnancy was established [18,19,20]. A further 4% of cows experienced abortion between 60 and 260 days of pregnancy. Approximately 75% of losses during the first 120 days of gestation were detected by the pedometer system as cows in estrus. In addition, 6% of pregnant cows showed estrus signs. Pregnant cows that showed signs of estrus on the day of the visit were included in the study (*n* = 12). For each of these cows, another cow previously diagnosed as pregnant and that at estrus no longer showed signs of pregnancy was also included (*n* = 12).

#### 2.4.3. Pregnancy Test Values 

**Figure 1:**
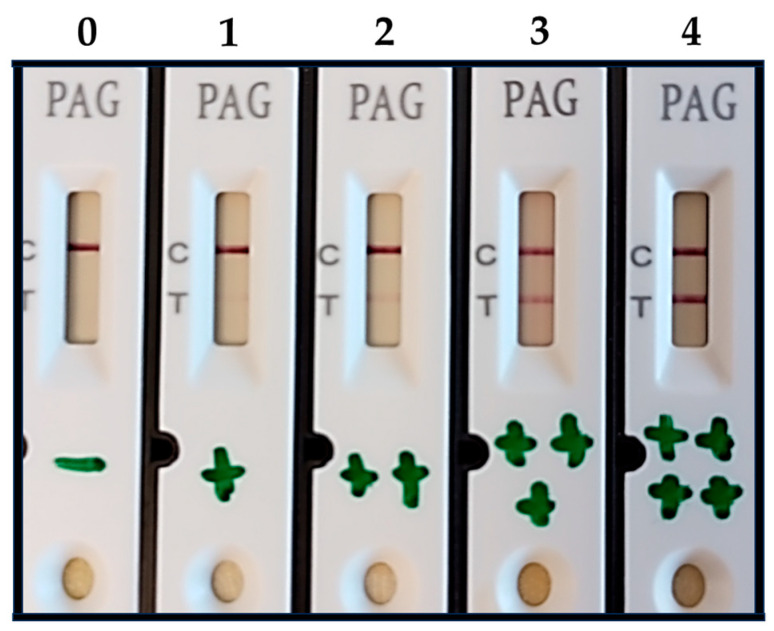
Photograph of the on-farm pregnancy test of plasma from five cows. Absence of the test line (T) indicated that the cow was not pregnant and the sample was categorized as “0”. The test line at any intensity indicated pregnancy and ranged from very light, categorized as “1”, to dark, or similar to the control line (C), categorized as “4”. Bovine serum albumin, bovine IgG, bovine casein, progesterone, estradiol, LH, and FSH had no cross-reactions with the test kit.

As the intensity of the test line ranged from very light to similar to the control line, four values (1, 2, 3, or 4) were given to the positive tests (Figure 1). Absence of test line, or value 0, indicated that the cow was not pregnant.

#### 2.4.4. Study Population 

The final study population constituted of 284 cows, involving a whole blood sample plus a plasma sample from each cow: 60 non-inseminated postpartum cows, 212 pregnant cows with 100 or more days in milk, and 12 non-pregnant cows (Table 1). Cows were included only once in the study.

### 2.5. Data Collection and Statistical Analyses

Ultrasound pregnancy results were used as the reference and compared to the whole blood and plasma PAG test results. The following data were recorded from each cow: parturition and AI dates, days in milk, and milk production (mean production in the previous seven days) at the time of the test; in pregnant cows: pregnancy days, pregnancy loss during the three weeks following a positive pregnancy diagnosis with a live conceptus, and presence of twins or a dead conceptus; and results of the test: positive or negative, and the test values (from 0 to 4). Results of the PAG tests were categorized as follows: diagnosis pregnant correct (a); diagnosis pregnant incorrect (b); diagnosis not pregnant correct (c), and diagnosis not pregnant incorrect (d). From these values, the sensitivity, the specificity, the positive predictive value, and the negative predictive value of the pregnancy diagnosis in the postpartum period and early pregnancy (28–34 or 49–55 days post-AI) were calculated for both types of samples (Table 1).

In order to evaluate possible factors influencing the test line intensity variations in early pregnancy, multinomial logistic regression analyses were performed [54,55]. Line intensity was the dependent variable, classified into four ordinal levels (0, 1, 2, and 3), and period of pregnancy (late embryonic period vs. early fetal period), presence of twins (*n* = 28), presence of a dead conceptus (*n* = 15), subsequent pregnancy loss (*n* = 12), days in milk and milk production at the time of the test, and parity (primiparous vs. multiparous) as independent variables. The analyses for both whole blood and plasma samples were conducted using the software package PASW Statistics for Windows Version 18.0 (SPSS Inc., Chicago, IL, USA), chosen for its comprehensive tools for conducting multinomial logistic regression. The reference category was the value of maximum intensity for each group: 2 for whole blood samples (involving the values 0, 1, and 2) and 3 for plasma samples (involving the values 1, 2, and 3). For example, the statistical model analyzing the plasma samples estimated the effects of each independent variable on the likelihood of an observation falling into categories 1 or 2, compared to the reference category 3. To ensure the robustness of the analyses, diagnostics checks were performed, including tests for multicollinearity among independent variables and the assessment of model fit. The significance of the coefficients was evaluated using Wald chi-square tests. Odds ratios were calculated by exponentiating the regression coefficients. Significance was set at *p* < 0.05. Variables are expressed as the mean ± standard deviation (SD). When appropriate, possible differences were analyzed by the Student’s *t*-test (means) or by the chi-square test (proportions).

## 3. Results

Mean milk production, days in milk at the time of the test, and lactation number for all cows included in the study population were 54.2 ± 10.4 (25.2–79.9) kg, 141.7 ± 58 (60–421) days, and 3.5 ± 1.9 (1–8) lactations, respectively (mean ± SD; ranges between parentheses). Data from the group of early pregnant cows (28–34 days plus 49–55 days of gestation) were 55.5 ± 10.4 (25.2–79.9) kg, 146.9 ± 48.3 (100–323) days, and 3.6 ± 1.8 (1–8) lactations. 

Table 1 shows sensitivity, specificity, positive predictive value, and negative predictive value by group (postpartum cows, cows in estrus, and pregnant cows) for whole blood and plasma samples. Specificity was 100% from Day 90 postpartum for blood samples and from Day 100 for plasma samples. The cut-off was 100 days in milk for the remaining samples. During the postpartum period, the test line intensity value in positive samples was 1 for whole blood and from 1 to 3 for plasma. Specificity was 100% for both blood and plasma samples from previously pregnant cows showing estrus: three from 42 to 59 days post-AI and nine from 108 to 180 days post-AI. Pregnancy loss was not detected in these cows before estrus. Sensitivity was 100% for blood and plasma samples from 116-to-200-days-pregnant cows showing estrus, with a test line intensity ranging from 2 to 4 in both types of samples. Sensitivity for whole blood samples was 75% in the late embryonic period and 76% in the early fetal period, whereas a greater sensitivity (100%: *p* < 0.0001) was registered for plasma samples in both periods. Mean test intensity values from whole blood samples were 0.8 ± 0.5 (0–2) and 0.8 ± 0.6 (0–2) units for the late embryonic period and early fetal period, respectively, whereas test values from plasma samples were 1.6 ± 0.7 (1–3) and 1.7 ± 0.7 (1–3) units for the same periods. Grouping samples from the late embryonic period and the early fetal period, intensity values from blood and plasma samples were: for twin pregnancies, 1.0 ± 0.5 (0–2) and 1.9 ± 0.8 (1–3) units; for pregnancies with a dead conceptus, 0.6 ± 0.5 (0–2) and 1.6 ± 0.6 (1–3) units; and for cows experiencing subsequent pregnancy loss, 0.6 ± 0.6 (0–2) and 1.6 ± 0.6 (1–3), respectively.

Based on the multinomial logistic regression procedures, no factors could be associated with the test line intensity values from the whole blood samples. Table 2 shows the results from the multinomial logistic regression analysis for the plasma samples. A significant relationship between milk production and line intensity results was found. For each additional kg of produced milk, the odds for plasma classified as category 1 versus the reference category 3 increased by a factor of 1.103, whereas the odds for plasma classified as category 2, as opposed to category 3, increased by a factor of 1051. The independent variables period of pregnancy, presence of twins, presence of a dead conceptus, subsequent pregnancy loss, days in milk, and parity had no influence on the test line intensity results.

As milk production did not influence the test line intensity values from the whole blood samples, mean milk production of early pregnant cows with negative results were compared to those cows with positive results using the Student’s *t*-test. Cows tested as negative (*n* = 50) had a significantly greater milk production than positive cows (*n* = 150): 62.1 ± 6.6 and 53.3 ± 8.4 kg, respectively (mean ± SD; *p* < 0.0001). 

## 4. Discussion

The current study shows the use of a newly developed pregnancy rapid test on whole blood and plasma for PAG detection and early pregnancy diagnosis in high-producing dairy cows. Points of this study to highlight would include: (a) the sensitivity of pregnancy diagnosis using plasma samples was 100% during the late embryonic and early fetal period, from days 28 to 55 of gestation; (b) the intensity of the test line in positive plasma samples during this period was related to milk production; (c) the proportion of blood samples showing undetectable PAG levels and false negative diagnoses was associated with high milk production; (d) twin pregnancies, presence of a dead conceptus, and subsequent pregnancy loss could not be differentiated from single pregnancies carrying a single live conceptus; (e) the test was useful at checking the presence of PAGs in cows previously diagnosed as pregnant and showing estrous signs from Day 40 to 200 post-AI, using both blood and plasma samples; and (f) specificity was 100% from day 90 postpartum for blood samples and from day 100 for plasma samples. 

False negative diagnosis of pregnant cows was 0% using plasma samples in a valuable population of 212 lactating cows, in contrast with previous studies performed with rapid tests on-farm [41,42,43]. An explanation for this discordance may be the fact that cows with less than 100 days in milk were not included in the present study. The incidence of false positive diagnoses between 80 and 100 days postpartum described here agrees with those previous studies using radioimmunoassay (RIA) methods [29,31,32]. As expected, due to dilution of whole blood samples with sample buffer, the use of blood samples was associated with a correct postpartum negative diagnosis earlier and with a sensitivity at the time of pregnancy diagnosis lower than plasma samples. The use of blood serum was also demonstrated to be more accurate and suitable than that of whole blood during early pregnancy in previous research [56]. However, the same results were observed using blood or plasma in advanced pregnancies, from Day 116 to 200 of gestation, a period with an important and progressive production of PAGs [28,29,30].

Categorization of test line intensity proved valuable in identifying possible factors influencing test results. High milk production was associated with a tenuous test line for plasma samples and a negative result for blood samples, in agreement with previous findings using RIA [40] or enzyme-linked immune sorbent assay (ELISA) [41], in which milk production correlated negatively with detection of PAGs. Surprisingly, the period of early pregnancy, late embryonic period (28–34 days of gestation) vs. early fetal period (49–55 days of gestation), had no influence on results. Although plasma PAG concentrations progressively increase from the peri-implantation period, between 18 and 22 days of gestation, through gestation, up to a few days before parturition [27,28,29,30], a significant decline from 35 to 56 days of gestation described using RIA [40], or from 30 to 60 using ELISA [41,57,58], could explain the similar test line values for both periods and, so, the lack of effect of time point analyses. From Day 60 of gestation, a clear significant positive effect of day of gestation on plasma PAG concentrations has been extensively assumed [27,28,29,30]. In effect, blood and plasma samples from advanced pregnant cows (116 to 200 days of gestation) included in this study showed a sensitivity of 100%, with a high test line intensity in both types of samples.

The plateau behavior of PAG levels detected during the transition from the late embryonic period to the early fetal period, together with large individual variations, may have favored the non-detection of twins, dead conceptus, or live conceptuses that experienced subsequent pregnancy loss. In contrast, checking cows previously diagnosed as pregnant and showing signs of estrus was helpful. With both types of samples, sensitivity and specificity were 100% in these cows. The manifestation of estrus in pregnant cows is frequent, with variable incidence among herds. Pregnant cows stood willingly to be mounted by another cow or bull at all stages of gestation, although more often in the middle of the gestation period [59,60]. In fact, more than 40% of cows with high milk progesterone levels may be inseminated [61] and 7% of pregnant cows were submitted to re-insemination in an extensive study [62]. The test made it possible to confirm with great certainty whether the cow was in estrus or pregnant.

## 5. Conclusions

The use of plasma samples was more effective than the use of whole blood samples in the diagnosis of early pregnancy. However, because of large individual variations, normal single pregnancies could not be differentiated from twin pregnancies, from pregnancies with a recent dead conceptus, nor from pregnancies with live conceptuses experiencing subsequent failure. In the case of cows previously diagnosed as pregnant and showing signs of estrus, both types of samples showed the same results. False positives in the postpartum period and the incidence of pregnancy loss during the late embryonic/early fetal period should be considered limitations of the test. These results taken together suggest that a positive pregnancy diagnosis should be confirmed a few days later.

## Figures and Tables

**Table 1:** Diagnosis of pregnancy in high-producing dairy cows by detecting pregnancy-associated glycoproteins (*n* = 284 lactating cows).

Categories of the Results ^(a)^	a	b	c	d	Sensitivity	Specificity	Positive Predictive Value	Negative Predictive Value
Postpartum ^(b)^								
Whole blood								
Days 60 to 69	0	6	4	0	0	40	0	100
Days 70 to 79	0	3	7	0	0	70	0	100
Days 80 to 89	0	2	8	0	0	80	0	100
Days 90 to 99	0	0	10	0	0	100	0	100
Days 100 to 109	0	0	10	0	0	100	0	100
Days 110 to 119	0	0	10	0	0	100	0	100
Plasma								
Days 60 to 69	0	10	0	0	0	0	0	0
Days 70 to 79	0	7	3	0	0	30	0	100
Days 80 to 89	0	4	6	0	0	60	0	100
Days 90 to 99	0	4	6	0	0	60	0	100
Days 100 to 109	0	0	10	0	0	100	0	100
Days 110 to 119	0	0	10	0	0	100	0	100
Cows in estrus								
Blood and plasma								
Open ^(c)^	0	0	12	0	0	100	0	100
Pregnant ^(d)^	12	0	0	0	100	0	100	0
Pregnant cows ^(e)^								
Whole blood								
28–34 days	75	0	0	25	75 *	0	100	0
49–55 days	76	0	0	24	76 *	0	100	0
Plasma								
28–34 days	100	0	0	0	100 **	0	100	0
49–55 days	100	0	0	0	100 **	0	100	0

^(a)^ Categories of the PAG test results were: a: diagnosis pregnant correct; b: diagnosis pregnant incorrect; c: diagnosis not pregnant correct; d: diagnosis not pregnant incorrect. Sensitivity = 100 × a/a + d. Specificity = 100 × c/c + b. Positive predictive value = 100 × a/a + b. Negative predictive value = 100 × c/c + d [52,53]. ^(b)^ Non-inseminated cows. ^(c)^ Cows diagnosed as pregnant from 12 to 95 days before estrus. ^(d)^ Pregnant cows showing signs of estrus. ^(e)^ Values with different superscripts within columns denote significant differences detected by the chi-square test (*, **: *p* < 0.0001).

**Table 2:** Multinomial logistic regression analysis of the test line intensity values from plasma samples in the early pregnancy (*n* = 200 lactating cows).

Milk Production	Coefficient	Standard Error	Odds Ratio	95% Confidence Interval	*p*
Test values from 3 to 1	0.098	0.023	1.103	1.054–1.55	<0.001
Test values from 3 to 2	0.05	0.022	1.051	1.007–1.097	0.023

## Data Availability

Data available on request due to restrictions (privacy). The data are not publicly available due to privacy and 3rd parties’ agreements.
